# Supplementary material for: Association between oral microbiome and seven types of cancers in East Asian population: a two-sample Mendelian randomization analysis
Source: Front Mol Biosci. 2023 Nov 21;10:1327893. doi: 10.3389/fmolb.2023.1327893 (PMC10702768; doi:10.3389/fmolb.2023.1327893)
Supplement: Supplementary file 1 [file DataSheet1.ZIP › Supplementary Materials/MR plots of saliva/saliva═╝/ovarian cancer/pheno.3352_to_ovarian cancer_leave_one_out.pdf]

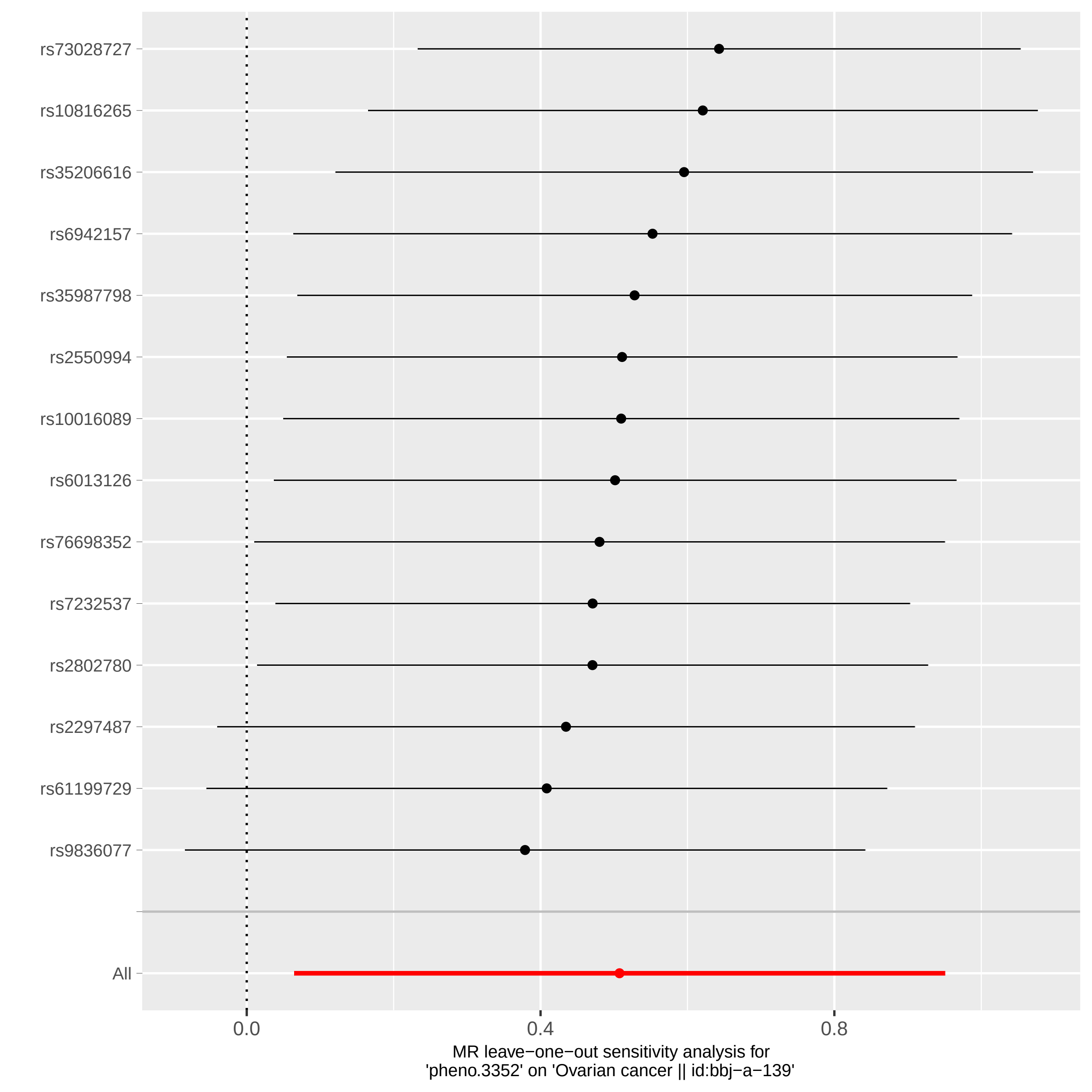
rs73028727
rs10816265
rs35206616
rs6942157
rs35987798
rs2550994
rs10016089
rs6013126
rs76698352
rs7232537
rs2802780
rs2297487
rs61199729
rs9836077
All
0.0
0.4
0.8
MR leave−one−out sensitivity analysis for
'pheno.3352' on 'Ovarian cancer || id:bbj−a−139'
